# Safety and Effectiveness of Combination Antiretroviral Therapy during the First Year of Treatment in HIV-1 Infected Rwandan Children: A Prospective Study

**DOI:** 10.1371/journal.pone.0111948

**Published:** 2014-11-03

**Authors:** Philippe R. Mutwa, Kimberly R. Boer, Brenda Asiimwe-Kateera, Diane Tuyishimire, Narcisse Muganga, Joep M. A. Lange, Janneke van de Wijgert, Anita Asiimwe, Peter Reiss, Sibyl P. M. Geelen

**Affiliations:** 1 Kigali University Teaching Hospital, Department of Pediatrics, Kigali, Rwanda; 2 Department of Global Health and Amsterdam Institute for Global Health and Development, Academic Medical Center, Amsterdam, The Netherlands; 3 Institute of Infection and Global Health, University of Liverpool, Liverpool, United of Kingdom; 4 Rinda Ubuzima, Kigali, Rwanda; 5 Wilhelmina Children's Hospital, University Medical Centre Utrecht, Utrecht, The Netherlands; 6 Biomedical Research, Epidemiology Unit, Royal Tropical Institute, Amsterdam, The Netherlands; 7 Outpatients Clinic, Treatment and Research on HIV/AIDS Centre, Kigali, Rwanda; 8 Ministry of Health of Rwanda, Kigali, Rwanda; London School of Hygiene and Tropical Medicine, United Kingdom

## Abstract

**Background:**

With increased availability of paediatric combination antiretroviral therapy (cART) in resource limited settings, cART outcomes and factors associated with outcomes should be assessed.

**Methods:**

HIV-infected children <15 years of age, initiating cART in Kigali, Rwanda, were followed for 18 months. Prospective clinical and laboratory assessments included weight-for-age (WAZ) and height-for-age (HAZ) z-scores, complete blood cell count, liver transaminases, creatinine and lipid profiles, CD4 T-cell count/percent, and plasma HIV-1 RNA concentration. Clinical success was defined as WAZ and WAZ >−2, immunological success as CD4 cells ≥500/mm^3^ and ≥25% for respectively children over 5 years and under 5 years, and virological success as a plasma HIV-1 RNA concentration <40 copies/mL.

**Results:**

Between March 2008 and December 2009, 123 HIV-infected children were included. The median (interquartile (IQR) age at cART initiation was 7.4 (3.2, 11.5) years; 40% were <5 years and 54% were female. Mean (95% confidence interval (95%CI)) HAZ and WAZ at baseline were −2.01 (−2.23, −1.80) and −1.73 (−1.95, −1.50) respectively and rose to −1.75 (−1.98, −1.51) and −1.17 (−1.38, −0.96) after 12 months of cART. The median (IQR) CD4 T-cell values for children <5 and ≥5 years of age were 20% (13, 28) and 337 (236, 484) cells/mm^3^respectively, and increased to 36% (28, 41) and 620 (375, 880) cells/mm^3^. After 12 months of cART, 24% of children had a detectable viral load, including 16% with virological failure (HIV-RNA>1000 c/mL). Older age at cART initiation, poor adherence, and exposure to antiretrovirals around birth were associated with virological failure. A third (33%) of children had side effects (by self-report or clinical assessment), but only 9% experienced a severe side effect requiring a cART regimen change.

**Conclusions:**

cART in Rwandan HIV-infected children was successful but success might be improved further by initiating cART as early as possible, optimizing adherence and optimizing management of side effects.

## Introduction

There is strong evidence that combination antiretroviral therapy (cART) reduces morbidity and mortality, promotes normal growth and development, and improves quality of life in children infected by HIV [1], [2], [3], [4], [5], [6]. However, cART effectiveness depends on durable suppression of viral replication. Ongoing HIV replication leads to chronic inflammation, and when cART is not used appropriately this can lead to HIV drug resistance and treatment failure, which limits future treatment options [7], [8]. Data from low and middle-income countries (LMIC) have demonstrated good cART effectiveness and tolerability in most children, but some children remain underweight and stunted or do not improve their CD4 T-cell count or viral load after several years of treatment [9], [10], [11]. Advanced disease at cART initiation was found to be associated with poor outcomes [9], [12], [13], [14], indicating that earlier treatment may improve effectiveness of cART. Initiation of cART in children is guided by pediatric clinical staging and age-dependent CD4 values.

In Rwanda the national ART guidelines were recently revised to promote an earlier start of cART in children and adolescents, and a roll out of pediatric care and treatment centers throughout the country was achieved [15]. As a result, the number of HIV-infected children on cART in Rwanda has rapidly increased from 468 in 2005 to an estimated 8,032 in 2013 [16]. The main objectives of this study were to prospectively document responses to cART in the first year of treatment in a cohort of HIV-infected Rwandan children, and to determine the incidence and severity of side effects of cART.

## Methods

### Ethical considerations

The Rwanda National Ethics Committee (RNEC) and the Medical Ethics Review Committee of the University Medical Center of Utrecht, the Netherlands, approved the study protocol. In accordance with the RNEC guidelines written informed consent was obtained from primary caregivers of all children. In addition, verbal assent was obtained from children between 7 and 12 years of age, and written assent from children age 12 or older. The Rwandan national guidelines for disclosure to children recommend to inform children at 7 years of age of their HIV status.

### Study design, population and period

In this longitudinal prospective cohort study, HIV-infected cART-naïve children below 15 years of age who initiated cART between March 2008 and December 2009 were followed by the study team for a minimum of 9 and a maximum of 18 months. Study participation ended after 18 months of follow-up or in September 2010, when funding for the study ended. All children continued to be followed in routine HIV care at a public clinic after their study participation ended. The study was conducted at the Treatment and Research AIDS Center (TRACplus) Outpatient Clinic in Kigali, Rwanda. During the study period, the TRACplus clinic was providing HIV care and treatment to 686 HIV-infected children. Among these children, 444 (65%) were already on cART before the study period, 174 became eligible for treatment. With the strategy to scale up pediatric treatment services, 51 children were transferred to clinics closer to their homes, hence they were not enrolled for the study. One hundred and twenty three (18%) children were enrolled. These children were usually referred from Kigali University Teaching Hospital (which is adjacent to the TRACplus clinic), nearby district hospitals, or health centers providing Prevention of Mother to Child HIV Transmission (PMTCT) services; a few children were diagnosed at the TRACplus facility itself. All children below the age of 15 years who initiated cART at the TRACplus clinic during the study period were given the opportunity to enroll in the study.

### cART guidelines and regimens

At the time of study initiation, the 2007 Rwandan ART guidelines (based on the 2006 WHO ART guidelines) were operational, which recommended cART initiation in children and adolescents less than 15 years of age if they were classified as WHO pediatric clinical stage III or IV, or had a severe immunodeficiency based on age-dependent CD4 values: CD4 <1500/mm^3^ or <25% if ≤11 months; <750/mm^3^ or <20% if 12–35 months; <15% or <350/mm^3^ if 36–59 months; and <350/mm^3^ if ≥5 years of age [17], [18]. Children enrolled in the study received cART, cotrimoxazole prophylaxis, and free medication for all acute illnesses during the length of the study. They were initiated on a first-line cART regimen consisting of two nucleoside reverse transcriptase inhibitors (NRTIs) and a non-nucleoside reverse transcriptase inhibitor (NNRTI). A cART regimen was defined as nevirapine-based, efavirenz-based or protease-inhibitor (PI)-based. The Rwandan ART guidelines were revised in 2009, and from then onwards, children known to have been exposed to nevirapine in the context of PMTCT were initiated on a first-line regimen with two NRTIs and a protease inhibitor (PI) [19]. For the purposes of this study, a treatment switch was defined as modifying the regimen to another regimen, within the first-line (including modification from one NRTI to another) or from first-line to second-line. Children would typically switch from a nevirapine-containing regimen to an efavirenz-containing regimen if side effects occurred due to nevirapine, or if they developed tuberculosis. A treatment switch from one NRTI to another NRTI could be due to national ART guidelines changes, side effects, or stock outs. A modification from a first line (NNRTI-containing) regimen to a second line (PI-containing) regimen was indicated in case of virological failure.

### Clinic procedures

By the time a child and caregiver were approached for study participation, the decision to start cART had already been taken by a committee of clinicians and social workers as per routine clinic procedures. Children who subsequently also consented to study participation initiated cART at study enrollment. At this enrollment visit, primary caregivers and children were counseled and interviewed by study staff. The face-to-face interviews included questions about socio-demographics (including orphan status and guardianship), HIV infection history, and variables deemed of importance in the context of cART adherence (see below). In accordance with the national ART guidelines, a clinical assessment was conducted at enrollment and at 2, 4, 8 and 12 weeks after enrollment, and subsequently every three months if the child was clinically doing well, until a maximum of 18 months of follow up was reached or the end of the study (whichever came earlier). Children had additional visits to the clinic in case of unforeseen problems (e.g. infections, or presumed adverse effects of cART). The study was conducted within the public health sector; clinic visits combined routine follow-up procedures and additional study-related procedures such as close laboratory monitoring including CD4 and viral load as well as treatment adverse effect assessment. The study was designed in such a way that no extra visits were needed for the sake of the study. For the study a reimbursement of transport fees was given to the parents per visit for the equivalent of 5 USD. The assessment included a physical examination with measurement of height and weight, pediatric WHO clinical staging, clinical symptoms, and targeted evaluations of side-effects using a standardized checklist. A general physical exam was also conducted and clinician findings were recorded on a standardized CRF covering each body system. In addition to the clinical and laboratory evaluations targeting side effects (see below), side effects were also assessed by standardized face-to-face interview at each study visit. In addition, participants were asked if they had any other symptoms that had not yet been covered in the interview.

### Laboratory testing

All laboratory tests were performed at the National Reference Laboratory (NRL) in Kigali, Rwanda. Blood was drawn by venipuncture: a complete blood cell count and liver transaminases [Alanine Amino Transferase (AST) and Aspartate Amino Transferase (AST)] were determined by Cobas Integra 400 plus (Roche Diagnostics, Indianapolis IN, USA) at enrollment and at 1, 3, 6, 12, and 18 months follow-up. CD4 T-cell counts and percentages were determined at enrollment and at 3, 6, 12 and 18 months follow-up by flow-cytometric measurement using a FACS Calibur (Becton Dickinson, San Jose, CA, USA). Plasma HIV-1 RNA concentration (Roche Cobas AmpliPrep/Cobas TaqMan HIV-1, Roche Molecular Systems, France, with a lower limit of detection of 40 copies/mL), creatinine (Cobas Integra 400 plus, Roche Diagnostics, Indianapolis IN, USA) and a lipid profile (low-density lipoprotein, high-density lipoprotein and triglycerides, Human Humastar 180, Human GmbH, Wiesbaden Germany) were determined at enrollment and at months 6, 12 and 18. Children with virological failure (see definition below) and children with plasma HIV-RNA concentrations between 40 and 1000 copies/mL were scheduled for additional HIV-1 RNA testing within 6 months.

### Adherence assessments

Caregivers and children, if age appropriate, received adherence counseling before enrollment and then at each follow-up visit. Caregivers were requested to return all medication containers and any unused medications at the next scheduled visit. For adherence monitoring, the caregivers were asked questions by face-to-face interviewing using a structured questionnaire; adherence assessment was conducted at every clinic and pharmacy follow-up visit.

They were asked how many doses of the prescribed medication the child had missed during the previous 30 days and at what time points this occurred, and reasons for non-adherence, both child-related (e.g. refusal, spitting, or vomiting) or caregiver-related (e.g. forgetting). They were also asked questions about the socio-economic status of the household (level of caregivers' education, household income), and distance to the clinic. Children were categorized as non-adherent if having taken less than 95% of the prescribed medication in the last 30 days. In addition, study nurses and pharmacy staff counted pills dispensed and returned unused, assuming that all other pills were used.

### Statistical analysis

All statistical analyses were performed using STATA Version 12 (Copyright 1984–2007 StataCorp TX USA). All statistical analyses were assessed for statistical significance at the p<0.05 level. Descriptive statistics are presented as proportions for categorical data and means with standard deviations (SD) and medians with IQR for parametric and non-parametric continuous data, respectively.

#### Study endpoints

The primary objective of this study was to determine the proportion of children achieving good clinical, immunological, and virological outcomes in the first year of cART as well as predictors of these outcomes. Good clinical outcome was defined as weight-for-age (WAZ) or height-for-age (HAZ)≥−2 z-score. WAZ and HAZ were calculated using Epinfo version 3.5.1 (Centers for Disease Control and Prevention, Atlanta, GA). Immunological success was defined as achievement of CD4 cells ≥500/mm^3^ for children above 5 years of age and a CD4 percentage ≥25% for children under 5 years of age. Virological success was defined as an HIV-1 RNA concentration <40 copies/mL per study time point. Children were categorized as WHO clinical stage I–IV throughout the study according to the WHO pediatric clinical classification system [18].

A secondary objective of the study was to document the occurrence and severity of side effects at any time point as well as predictors of the occurrence of side effects. Self-reported side effects and clinical findings were categorized into 5 main groups: gastro-intestinal, neurological, skin/mucosal, respiratory, and other. The severity of each side effect was assessed using the US National Institute of Allergy and Infectious Diseases Division of AIDS Table for Grading the Severity of Adult and Pediatric Adverse Events (DAIDS-AE) [20]. Grade 1 (mild) was defined as symptoms causing no or minimal interference with usual social and functional activities; grade 2 (moderate) as symptoms causing greater than minimal interference with usual social and functional activities; grade 3 (severe) as symptoms causing inability to perform usual social and functional activities; and grade 4 (potentially life-threatening) as symptoms causing inability to perform basic self-care functions or medical or operative intervention indicated to prevent permanent impairment, persistent disability, or death. Side effects were classified as transient if they were recorded at one or multiple time-points but eventually disappeared without any changes to cART regimen and without treatment of the side effects. They were classified as persistent when they required cART regimen changes or treatment of the side effect. Children were considered to have severe anemia if hemoglobin was <7.5 g/dl, and severe liver abnormality if ALT and/or AST was >5 times the normal values.

Another secondary objective of the study was to assess adherence over time and predictors of adherence. Children were categorized as poorly adherent if they had taken less than 95% of the prescribed medication, based on either self-report or pill counts, or if the caregiver had missed a scheduled pharmacy appointments for ≥2 consecutive days, as only a 15- or 30-day supply of medication was provided at each clinic visit. Adherence assessment was conducted at every visit and was measured for the last 30 days preceding the clinic visit.

#### Statistical modeling

Due to repeated measurements of the outcome, generalizing estimating equation (GEE) models were used to determine outcome changes over time, assuming an exchangeable correlation, (where the correlation is the same for all outcomes within a subject; which is best suited for longitudinal studies in which the same subjects are followed over time). The associations between outcomes and different explanatory variables were evaluated using bivariable GEE models (due to sample size limitations, only one explanatory factor was added at a time). In the WAZ and HAZ models, these explanatory variables included age group, CD4 count, gender, PMTCT exposure and adherence at visits 3, 6 and 9. In the models with positive immunological response as the outcome, children of all ages were combined into one model by combining percentages ≥25% for children below 5 years and absolute CD4 T-cell counts ≥500 cell/mm^3^ for children older than 5 years of age as positive outcomes. Explanatory variables included age group, CD4 count, WHO stage, gender, PMTCT exposure and adherence during the previous 3 or 6 months. In the models with virological success as the outcome, the same explanatory variables were tested as in the immunological success models, but also distance to the TRACplus clinic, orphan status, caregiver education, viral load at initiation and history of treatment switches. Furthermore, adherence over the last 6 months was used (instead of the last 3 months in all other models) because viral load was only measured once every 6 months. In the models with adherence over time as the outcome, explanatory variables included cART regimen, gender, caregiver's educational level, orphan status, and distance to the TRACplus clinic. The proportions of children with side effects were calculated per time point. From the literature, the most relevant predictors associated with side effects were considered gender and age [21]. Using Kaplan–Meier survival analysis, gender and age were analyzed for all side effects jointly and for each group separately.

## Results

### Baseline characteristics

**Figure 1:**
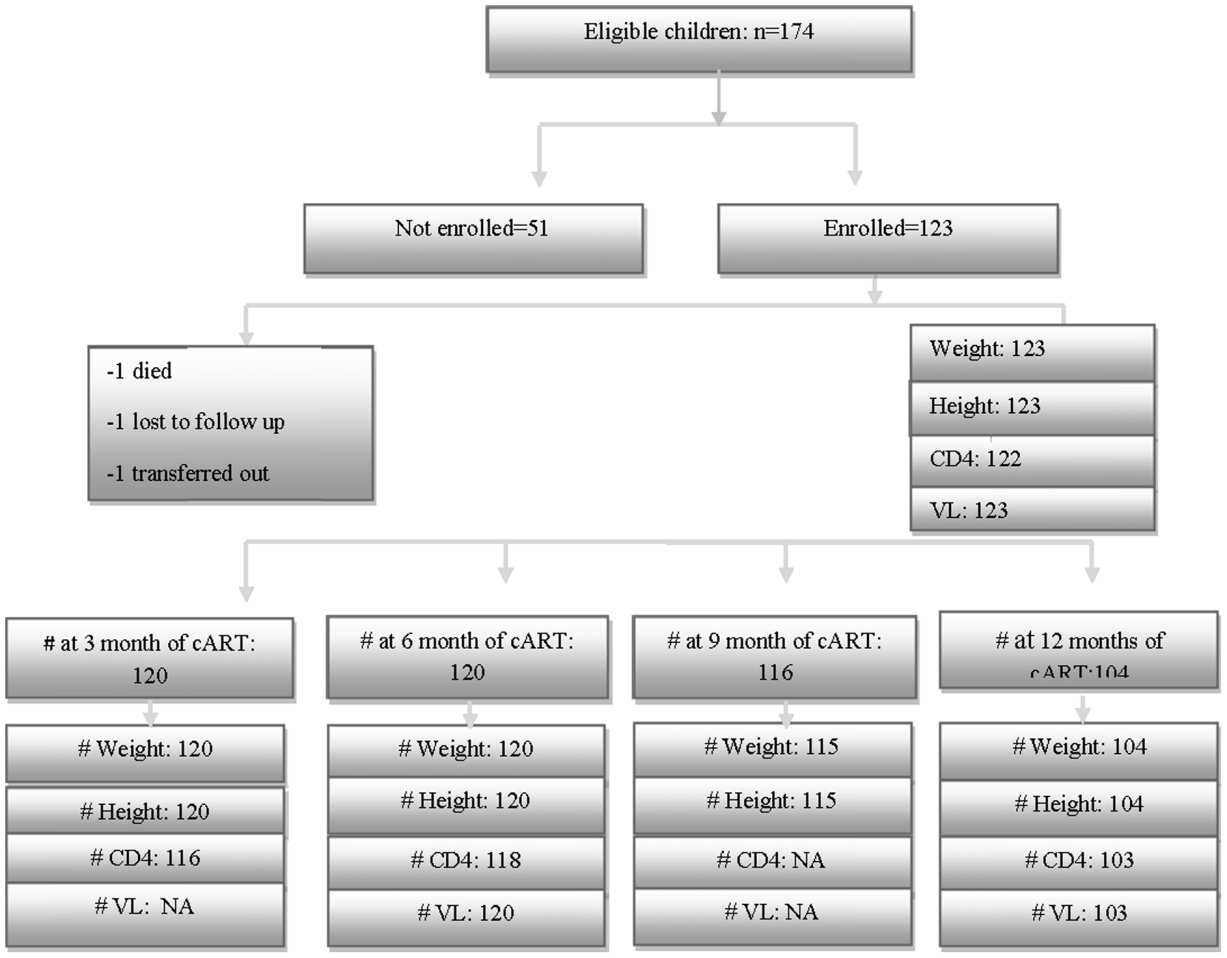
Flowchart summarizing the number of children at each stage of the study.

One hundred and twenty three children were enrolled in the study (figure 1). The median (IQR) age at cART initiation was 7.4 years (3.2, 11.5); 40% of children were below 5 years of age and 54% were female (Table 1). Twenty-five (20%) children were diagnosed with HIV during PMTCT follow-up services, 58 (47%) children when they presented with clinical symptoms, and 40 (33%) children after their parents or siblings were diagnosed with HIV or were suspected to have died of HIV-related diseases. More than a quarter of the children (26%) were orphaned and cared for by other family members or living in an orphanage.

**Table 1:** Baseline characteristics at cART initiation (n = 123).

	<5 years (n = 49)	≥5 years (n = 74)	N
**DEMOGRAPHIC AND SOCIAL**			
Median (IQR) age, years	2.5(1.8–3.5)	11.0(9.0–13.4)	**123**
Female n (%)	28(57)	39(53)	**123**
Perinatal infection n (%)	49(100)	74(100)	**123**
Children exposed to PMTCT, n (%)	15(33)	10(15)	**110**
**Parent status n (%)** *			**123**
Both parents alive	28(57)	34(46)	
Only mother alive	7(14)	12(16)	
Only father alive	4(8)	6(8)	
Both parents died	10(20)	22(30)	
**Guardians/caregivers n (%)**			**123**
Both parents	19(39)	20(27)	
Mother only	15(31)	25(34)	
Father only	2(4)	3(4)	
Other family member	11(23)	23(31)	
Other	2(4)	3(4)	
**Caregiver's educational status n (%)** **			**123**
No education/few years primary school	17(34.7)	28(37.85)	
At least primary school completed	32(65.3)	46(62.2)	
**Distance from healthcare center n (%)** ***			**123**
Living in Kigali	39(79.6)	16(21.6)	
Living outside of Kigali	10(20.4)	58(78.4)	
**Tested and Diagnosed n (%)**			**123**
PMTCT services	15	10	
Family member died/sick	15	25	
Symptoms	19	39	
**CLINICAL**			
**Weight-for-age z-score**			**123**
Mean (SD)	−2.04(1.6)	−1.49(1.1)	
z-score ≤−2 n (%)	26(53)	25(34)	
**Height-for-age z-score**			**123**
Mean (SD)	−2.5(1.4)	−1.7(0.9)	
z-score ≤−2 n (%)	34(69)	29(39)	
**WHO stage, n (%)**			**122**
Stage 1 & II	5(10.2)	18(24.7)	
Stage III & IV	44(89.8)	55(75.3)	
Tuberculosis at cART, n (%)	11(22)	19(26)	123
**LABORATORY**			
**Immunological status at baseline**			**119**
Median (IQR) CD4 values	20(13–28)	337/mm3(236–484)	
Children with CD4<15% or <350/mm3, n (%)	13(28)	41 (57)	
**Virological status at baseline**			**123**
Median (IQR) HIV-1 RNA copies/mL	283,000(59,800–1,100,000)	90,600(12,800–268,000)	
**Biochemistry & Hematology at baseline**			**122**
Median (IQR) ALT, UI/l	20(15–32)	19(15–25)	
Median (IQR) AST, UI/l	40(34–50)	34(29–40)	
Median (IQR) Hemoglobin, g/dL	11(10.2–11.6)	12(11.7–12.8)	
**INITIAL cART REGIMEN n (%)**			**123**
AZT/3TC/NVP	38(77.5)	46(62.2)	
ABC/3TC/NVP	1(2	2(2.7)	
D4T/3TC/NVP	2(4)	11(15.0)	
AZT/3TC/EFV	4(8)	12(16.2)	
D4T/3TC/EFV	2(4)	2(2.7)	
ABC/3TC/LPV/r	2(4)	1(1.4)	
Treatment switch	9(18.4)	12(16.2)	

*Orphan status was defined as having at least one biological parent vs. none.

**Caregiver's educational level was categorized as non-educated/few years of primary school vs. completed at least primary school.

***Distance to the clinic was defined as living in Kigali vs. outside of Kigali.

More children were stunted than underweight, with a mean (SD) HAZ and WAZ of −2.01 (1.2) and −1.73 (1.3), respectively. The median (IQR) CD4 T-cell values for children <5 years and ≥5 years of age were 20 (13, 28) percent and 337 (236, 484) cells/mm^3^ respectively. The median (IQR) plasma HIV-1 RNA concentration was 283,000 copies/mL (59,800, 1,100,000) for children less than 5 years of age and 90,600 copies/mL (12,800, 268,000) for those ≥5 years. The initial cART regimens are described in Table 1.

### Study follow-up and clinical outcomes

Due to termination of funding in September 2010, not all children could be followed for 18 months; 116 children had been followed for 9 months; 104 for 12 months and 72 children for 18 months. The longitudinal analysis includes all data up to 12 months. One child (14 years old) presenting with WHO clinical stage 4 and a CD4 T-cell count of 2 cells/mm^3^ died one month after cART initiation. He developed high fever, respiratory distress and increased lymphadenopathy, and the presumed cause of death was immune reconstitution inflammatory syndrome (IRIS). Two children were diagnosed with tuberculosis (one after 1 month on cART and the second child after 4 months of cART); no other children developed opportunistic infections during follow-up. One child was lost to follow-up, and one child moved and was transferred to another treatment center. At the time of the current analysis 98% of children were alive.

Table 2 summarizes anthropometric and biological parameters over time. Mean WAZ (GEE odds ratio (OR): 1.05 (95% confidence interval (CI): 1.03, 1.06; p-value <0.001)) and HAZ (GEE OR: 1.02 (95% CI: 1.01, 1.03; p-value <0.001)) improved significantly during the 12 months. At 12 months of follow-up, the proportion of children who were underweight had decreased from 42% to 20% (GEE OR: 0.90 (95% CI: 0.87, 0.94; p-value <0.001)) and the proportion of children who were stunted decreased from 51% to 41% (GEE OR: 0.96 (95% CI: 0.95, 0.99; p-value<0.001)). The median (range) ALT and AST concentrations at baseline were 19.5 (15.0, 25.0) and 39.0 (29.0, 50.0) IU/mL and rose to 22.0 (15.0, 30.0) and 54.1 (29.9, 91.9) IU/mL at 12 months, respectively. The median (range) hemoglobin at cART initiation was 11.6 (11.3, 12.0) g/dL and increased to 12.3 (12.1, 12.5) at 12 months of cART.

**Table 2:** Changes of WAZ, HAZ, Hemoglobin, CD4 values and HIV RNA overtime.

	Baseline (n = 123)	3 Months (n = 119)	6 Months (n = 119)	12 Months (n = 104)	P-values
**Nutritional status**					
WAZ (mean, 95%CI*)	−1.73(−1.95,−1.50)	−1.38(−1.59,−1.17)	−1.28(−1.47,−1.08)	−1.17(−1.38,−0.96)	**<0.001**
Underweight (WAZ<−2) (n, %)	52(42)	36 (30)	31(26)	21(20)	**<0.001**
HAZ (mean, 95%CI*)	−2.01(−2.23,−1.80)	−1.93(−2.15,−1.71)	−1.82(−2.04,−1.60)	−1.75(−1.98,−1,51)	**<0.001**
Stunting (HAZ<−2) (n, %)	63(51)	57(47)	53(44)	43(41)	**0.001**
Hemoglobin (mean, 95 CI*), g/dL	11.6(11.3–12.0)	12.1(11.8–12.4)	12.2(12.0–12.4)	12.3(12.1–12.5)	**<0.001**
**Immunological status**					
**Children ≥5 years, n = 74**					
CD4 T-cells (median, IQR)	337(236–484)	500(345–675)	567(365–765)	620(375–880)	**<0.001**
CD4 T-cells <500, n (%),	57(79)	34(49)	29(40)	22(34)	**<0.001**
**Children <5 years, n = 49**					
CD4 T-cell % (median, IQR)	20(13–28)	29(22–36)	33(26–39)	36(28–41)	**<0.001**
CD4T-cell <25%, n (%),	30(65)	17(38)	11(24)	5(13)	**<0.001**
**Virological status (HIV-RNA)**					
<40 (copies/mL), n (%)	0(0)	Not determined	52(43)	79(76)	**<0.001**
>1000 copies/mL, n (%)	123(100)		46(38)	17(16)	**<0.001**

**P values bold:** Changes over time for all variables were statistically significant at p<0.05 using a univariate Generalized estimating equation model for categorical, normal and no normal distributed outcomes.

Both age groups, above and below 5 years at cART initiation, had a significant increase in HAZ z-scores on therapy, but improvement was better in children above 5 years of age (GEE OR: 3.7 (95% CI: 1.8, 7.6)). Children who were not underweight when initiating cART were more likely to experience a significant HAZ and reduction of stunting overtime (GEE OR: 4.1 (95% CI: 1.9, 8.6)) as compared to those who were underweight (data not presented). WAZ increase was observed in both children with good and poor adherence, but the increase was slightly better in children with good adherence than children with poor adherence (GEE OR: 1.2 (95% CI: 0.9, 1.7)). Children who initiated treatment with CD4 ≥350 cells/µ/L or CD4 ≥15% had better improvement in WAZ scores compared to children who initiated treatment with CD4 below these cut offs (GEE OR: 1.9 (95% CI: 0.9, 3.8)). Children who achieved viral suppression compared to children with virological failure had significant WAZ (GEE OR: 2.3 (95% CI: 1.6, 3.3)) and HAZ increases over time (GEE OR: 1.3 (95% CI: 1.1, 1.5)). A small percentage of children did not show any improvement in WAZ (4%) and HAZ (9%) after 12 months of cART. Out of 5 children who did not have increased WAZ, three had virological and immunological failure, and 4 had poor adherence. Out of 12 who did not have increased HAZ, 9 had virological failure, 7 had immunological failure, and 7 had poor adherence.

### Immunological and virological responses

A significant increase in CD4 T- cells was observed in both children younger than 5 years of age as well as in those over 5 years of age. The median CD4 T-cell percent for younger children increased from 20% to 36% and the median CD4 T-cell count for children above 5 years increased from 337 to 620cells/mm^3^ by 12 months follow-up; the proportion of children who achieved immunological success was 87% for children under 5 years and 66% for children 5 years of age and above. In bivariable models, independent predictors of immunological success included age and CD4 T-cell baseline value, with children below 5 years and those having a CD4 T-cell baseline value above 350/mm^3^ showing a more robust increase in median CD4 T-cells (GEE OR: 1.9 (95% CI: 1.1, 4.1) and 7.0 (4.3, 11.6), respectively). PMTCT exposure, WHO stage at baseline, poor adherence, and socio-economic characteristics were not statistically associated with CD4 T-cell recovery in the first 12 months of cART.

The mean changes in HIV RNA plasma concentration over time are presented in Table 2. After 12 months of cART, 24% had detectable HIV RNA (>40 copies/mL), including 16% with virologic failure (>1000 copies/mL). In bivariable analyses, independent predictors of virological failure were being less than 5 years old at baseline (GEE OR: 2.6 (95% CI: 1.3, 5.2)), exposure to PMTCT (GEE OR: 3.4 (95% CI: (1.5, 7.8)), poor adherence during the first six months of treatment (GEE OR: 2.5 (95% CI: 1.3, 5.0)) and initiating cART with viral load ≥50.000 copies/mL (GEE OR: 4.6 (95% CI: 2.3, 156.2)). Other medical and social characteristics were not significantly associated with HIV RNA change over time (Table 3).

**Table 3:** Number of children with virological failure* over time.

	Month 6	Month 12	p-values**	Odds Ratio(95%%CI)
**Parent status**				
Both died	12(38)	6(21)	0.872	0.9(0.4–2.0)
At least one lives	34(39)	11(15)		
**Caregiver's educational status**				
Completed at least Primary school	17(37)	9(19)	0.678	0.6(0.3–3.1)
Non educated	30(39)	16(20)		
Distance from healthcare center				
Living in Kigali	22(40)	9(17)	0.567	0.8(0.5–2.7)
Outside of Kigali	28(41)	13(19)		
**Age group**				
Age ≥5 years at cART initiation	21(29)	7(11)	**0.006**	**2.6(1.3–5.2)**
Age <5 years at cART initiation	25(52)	10(25)		
**Immunological status at baseline**				
CD4≥15% or 350/mm3	21(34)	6(12)	0.282	1.5(0.7–2.9)
CD4<15% or <350/mm3	23(43)	10(20)		
**WHO stage at baseline**				
Baseline WHO I&II	8(35)	0	0.171	1.7(0.8–3.9)
Baseline WHO III&IV	37(39)	16(19)		
**95% Adherence**				
Adherent	26(32)	12(15)	**0.009**	**2.5(1.3–5.0)**
Non-adherent	20(53)	5(24)		
**PMTCT exposure**				
No-exposure	26(31)	9(12)	**0.003**	**3.4(1.5–7.9)**
Exposure	14(58)	6(38)		
**Regimen switch up to 6 or 9 months**				
No treatment change	40(37)	12(14)	0.337	1.6(0.6–4.3)
Treatment change	6(54)	7(49)		

*Virological failure defined as VL≥1000 copies/mL for one measurement;

**between group comparisons.

**P values bold:** significant difference at p<0.05.

### Adverse effects of treatment and treatment switches

**Figure 2:**
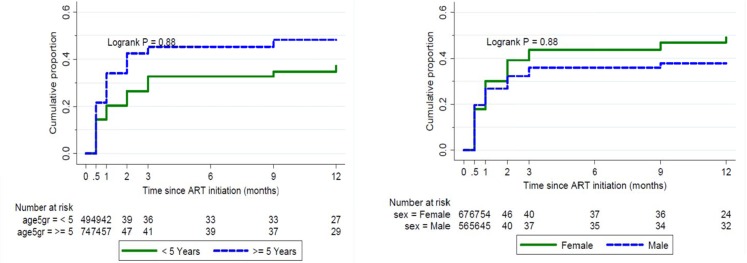
Cumulative of proportion of children with any side effect by sex (left) and age group (right) from baseline to 12 months of Treatment.

There were 158 cumulative adverse effects reported in 52 (42%) children during the period of 12 months. The highest number was reported at month two of treatment in 40 (33%) of the children. The majority (47 out of 52) of children reported the same adverse effect at 2 or more time points; 41 children had mild and transient adverse effects which recovered without stopping treatment, 11/123 (9%) children experienced persistent side effects and/or worsening over time and underwent cART regimen changes as a result. The incidence of side-effects was higher within the first 6 months of cART initiation than thereafter (Figure 2a, 2b). The most common side effects were nausea and vomiting (14.8%), nevirapine-associated skin rash and hypersensitivity (13.2%), any grade of anemia (7%), diarrhea (6%), and dizziness and fatigue (5%) (Table 4). Eighty six percent of mild/moderate side effects improved without additional therapy.

**Table 4:** Clinical and laboratory side effects.

	Day 15	Month 1	Month 2	Month 3	Month 6	Month 9	Month 12
**Clinical side effects, n (%)**							
***Skin, mucosa and nails***							
Hypersensitivity/Skin rash	16(13)	31(26)	26(21)	18(15)	6(5)	5(4)	2(2)
Nail pigmentation	-	-	-	2(2)	7(6)	9(8)	9(9)
***Gastro-intestinal***							
Nausea and vomiting, n (%)	21(17)	30(25)	34(28)	18(15)	4(3)	2(2)	1(1)
Diarrhea, n(%)	12(10)	13(11)	15(13)	11(9)	-	-	-
Abdominal pain, n (%)	4(3)	1(1)	2(2)	1(1)	-	-	-
***Neurological***							
Dizziness, n(%)	3(2)	6(5)	6(5)	6(5)	2(2)	2(2)	1(1)
Insomnia, n(%)	-	-	-	2(2)	2(2)	2(2)	1(1)
**Laboratory**							
Anemia (Hb<10 g/dl)	NA	NA	19(16)	6(5)	4(3)	2(2)	2(2)
Liver functions elevated (ALT), n (%)	7(6)	13(10)	8(7)	8(7)	3(3)	2(2)	-
**Other** * **, n (%)**	4(3)	2(2)	5(4)	3(2)	1(1)	-	-

*other included fatigue, anxiety and nightmares.

During the follow-up period of 18 months, 21 (17%) children switched their initial cART regimen. Reasons for switching included persistent side effects (n = 11), virological failure (n = 3), tuberculosis treatment (replacement of nevirapine by efavirenz, n = 2), replacement of stavudine following a change of the Rwandan cART guidelines (n = 3), and replacement due to stock-out issues (n = 2). The children who developed nevirapine-associated skin-related side effects and/or significant liver enzyme elevations stopped treatment and their symptoms abated after treatment cessation; they resumed treatment with an efavirenz-based regimen. In three children with significant anemia, zidovudine was replaced by abacavir or tenofovir, hemoglobin improved to ≥10 g/dl within 6 months after treatment change. Two adolescents on a stavudine-based regimen developed signs of lipoatrophy (n = 1) and lipohypertrophy (n = 1) during the second year of treatment, and switched from stavudine to tenofovir. Eighteen out of the 21 treatment modifications were from one drug to another within the first line; 3 children switched from the first line NNRTI based regimen to a second line with lopinavir/ritonavir because of virologic failure.

### Adherence assessment

Adherence as estimated by self-report was high, with a median >95% at all-time points. In only 77 (11%) out of 710 scheduled visits at least one missed dose was reported by self-report. Incorrect dosage of any drug or change of time of taking medication was reported in 44 (6%) of all scheduled visits. Poor adherence on self-report was highly predictive of poor adherence on pill count (data not shown); however, good adherence by self-report was often not confirmed by pill count data. Pill count data showed that pills or scheduled visits were missed at 269 (38%) of visits. The proportion of children who were poor adherent according to pill count and recorded missed visits (adherence <95%) decreased over time, from 38% at month 3 to 20% by 12 months.

In bivariable analysis, after 6 months of cART 48% of children with good adherence had an undetectable viral load while only 34% of children with poor adherence had an undetectable viral load. The proportion of children with an undetectable viral load increased to 80% in the adherent group and to 57% in the less adherent group after 12 months of cART.

In bivariable analysis, lower caregiver educational status (GEE OR: 1.2 (95% CI: (1.1, 9.8)) and being an orphan ((GEE OR: 1.6 (95% CI): (1.2, 8.3)) were associated with lower adherence. None of the other factors, including gender, age, regimen or distance to the clinic were found to be statistically associated with adherence over time.

## Discussion

The majority of children in this study showed good clinical and immunologic recovery, good adherence to cART and retention in care, as well as improved height and weight after 12 months of cART. Although the study showed overall treatment success after 12 months, HIV viral load was not fully suppressed in nearly a quarter of children, and immunologic recovery was less successful in children 5 years or older and those with more advanced HIV disease at cART initiation. Approximately one in 10 children developed severe side effects resulting in temporary cART cessation and regimen switches.

At baseline, slightly more than half of children were stunted and 40% were underweight. These proportions were higher than those reported by various recent national surveys in children regardless of HIV status, in which 27–44% of children were stunted, and up to 12% of children were underweight, depending on the area of the survey [22], [23]. The lowest proportion of underweight children (6%) was found in Kigali district [22]. However, in our study, the number of children stunted and underweight after 12 months of cART was close to these survey figures. Furthermore, the overall increase in weight among all children, and increase of height by baseline nutrition status and age are comparable to increases reported in other studies from similar resource constrained settings [9], [24], [25], [26], [27], [28], [29]. These findings reflect the efficacy of cART and offer reassuring evidence of its safety and tolerability in Rwandan children in which regular clinical monitoring is routine. Children who did not show any improvement in WAZ and HAZ after 12 months of treatment also showed poor ART adherence, poor immunologic recovery and virological failure.

In our study, children above 5 years of age were more likely to have impaired immunological recovery after 12 months of cART; conversely those under five were more likely to experience virologic failure. The robust immunologic recovery that we observed in young children may be partially explained by the superior ability of young infants to repopulate T lymphocytes [30], [31], or by the hypothesis that later initiation of cART allows for more architectural damage to lymphatic tissues, which in turn hampers immune reconstitution [4], [32]. The relationship between young age and virological failure has been documented in other studies in sub-Saharan Africa [13], [33]. It is also consistent with earlier observations that infants and young children often present with high viral load and that it takes longer to fully suppress viral replication [34], [35], [36], [37], [38]. Prospective studies with longer follow-up periods should be conducted to determine whether the association between age at cART initiation and suboptimal virologic suppression is maintained after a longer period on cART. Not surprisingly, age and immune status at cART initiation were the factors most strongly associated with immunological success [9], [39]. This observation emphasizes the fact that efforts should be made to diagnose and treat HIV infected children as early as possible.

The proportion of children with a detectable viral load and virological failure after 12 months of cART in this study is a serious finding that needs urgent attention. Poor adherence to cART and nevirapine exposure during PMTCT were associated with cART failure in our study as has been shown in other studies [40], [41]. Previous studies confirmed that one in two infants exposed to single dose nevirapine prophylaxis develop nevirapine resistance, which in turn may expose children to more resistant viral strains and compromise therapy with NNRTI-containing regimens [42], [43], [44], [45], [46], [47]. The finding that non-adherence measured by pill count was a strong predictor of virologic response reiterates the importance of adherence to cART and adherence monitoring through other means than self-report, in which patients are more likely to report high adherence [48]. Although it is obvious that adherence is paramount for achieving and maintaining viral suppression, and prevention of drug-resistance [49], [50], it seems that the specific challenges regarding adherence in pediatric populations have not yet been sufficiently tackled. Issues such as drug administration in young children, adolescent behavior, socio-economic status and the dependency on the caregiver [51], [52] may all play a role and are often difficult to solve. Additionally, the pharmacokinetic properties of ARVs in young children are not well known; underdosing or increased metabolism may lead to suboptimal plasma drug levels and thereby influence virologic outcomes. In an earlier study conducted in Rwanda, we observed that 14% of children using efavirenz were not adequately dosed [53], and other studies have shown similar results for other drug combinations [54], [55].

The incidence of mild and moderate side effects in this study, mainly associated with nevirapine use, were similar to the findings from Uganda by Tukei [56], but were higher than what was reported by Lapphra in Thai children [57] and by Oumar in Malian children [58]. Most of the severe symptoms reported were reversed after discontinuation of the suspected drug and treatment changes to potentially less toxic medication as has been reported previously by Shubber et al [59]. The number of children with severe and/or persistent side effects leading to drug substitution was higher in our study compared to the studies mentioned previously [56], [57], [58]. Side effects and treatment switches may impact on treatment adherence, and potentially undermine the success of cART [60], [61]. The side effects and regimen changes may have contributed to the relatively high percentage of children with treatment failure that we have seen in our study. More than half of the children without viral suppression had persistent side effects and/or regimen switches. Careful monitoring of the safety of cART remains needed, especially during the first months of cART, and we strongly support the national ART guidelines recommending PI-based regimens for all children <3 years of age [15].

This study has a number of limitations. Due to funding constraints, the sample size was relatively small; results for the present study on 123 children may not be generalizable to the larger population of children in Rwanda, given that data were only collected from children in one center in Kigali. Moreover, data for this study were collected a few years ago, current practice, validity and implication of some recommendations may be affected. However, the study presents more comprehensive information on cART outcomes in children than is available from national Rwandan HIV programs. The study highlights an important point that treatment failures are common in Rwandan children and emphasizes the importance of close virological monitoring. Another limitation is that the duration of follow-up was shorter than originally planned. This means that we were unable to assess the long term impact of cART on growth, immune and virologic responses, and long term toxicity. Studies with a longer duration of follow up are needed to inform national programs. Furthermore, we could not attribute the incidence of anemia solely to use of zidovudine as coexisting nutritional deficiencies and other chronic diseases may also have played a role [56], [62], [63]. Finally, we could not assess cART drug resistance in this particular study, but an earlier study in Rwanda showed that >90% of children with a viral load ≥1000 copies/mL after 12 months of cART were reported to have at least one NRTI or NNRTI's major mutations [10].

In conclusion, the importance of timely initiation of cART before profound immunodeficiency occurs in children should not be underestimated, as has been reported previously [9], [11], [12], [13], [14]. The Ministry of Health in Rwanda has recognized this and has adjusted its guidelines accordingly. Initiation of cART is now recommended for all children under 5 regardless of clinical condition and CD4 status, and for all children and adults older than 5 years with CD4 T-cells <500/mm^3^. However, several challenges related to side effects, and achieving long-term adherence and virologic suppression remain. To be truly successful, pediatric HIV programs must aim to find HIV infected children before disease progression occurs, initiate cART timely, and monitor treatment success and side effects closely. Furthermore, the main causes of virologic failure should be further investigated, so that strategies for early recognition of children at high risk and appropriate interventions can be developed [64], [65].
